# Overproduction of Phospholipids by the Kennedy Pathway Leads to Hypervirulence in *Candida albicans*

**DOI:** 10.3389/fmicb.2019.00086

**Published:** 2019-02-07

**Authors:** Robert N. Tams, Chelsi D. Cassilly, Sanket Anaokar, William T. Brewer, Justin T. Dinsmore, Ying-Lien Chen, Jana Patton-Vogt, Todd B. Reynolds

**Affiliations:** ^1^Department of Microbiology, The University of Tennessee, Knoxville, Knoxville, TN, United States; ^2^Department of Biological Sciences, Duquesne University, Pittsburgh, PA, United States; ^3^Department of Plant Pathology and Microbiology, National Taiwan University, Taipei, Taiwan

**Keywords:** *Candida albicans*, CDP-DAG pathway, *EPT1*, Kennedy pathway, *PEM1*, PEM2, phosphatidylcholine, phosphatidylethanolamine

## Abstract

*Candida albicans* is an opportunistic human fungal pathogen that causes life-threatening systemic infections, as well as oral mucosal infections. Phospholipids are crucial for pathogenesis in *C. albicans*, as disruption of phosphatidylserine (PS) and phosphatidylethanolamine (PE) biosynthesis within the cytidine diphosphate diacylglycerol (CDP-DAG) pathway causes avirulence in a mouse model of systemic infection. The synthesis of PE by this pathway plays a crucial role in virulence, but it was unknown if downstream conversion of PE to phosphatidylcholine (PC) is required for pathogenicity. Therefore, the enzymes responsible for methylating PE to PC, Pem1 and Pem2, were disrupted. The resulting *pem1*Δ/Δ *pem2*Δ*/*Δ mutant was not less virulent in mice, but rather hypervirulent. Since the *pem1*Δ*/*Δ *pem2*Δ*/*Δ mutant accumulated PE, this led to the hypothesis that increased PE synthesis increases virulence. To test this, the alternative Kennedy pathway for PE/PC synthesis was exploited. This pathway makes PE and PC from exogenous ethanolamine and choline, respectively, using three enzymatic steps. In contrast to *Saccharomyces cerevisiae*, *C. albicans* was found to use one enzyme, Ept1, for the final enzymatic step (ethanolamine/cholinephosphotransferase) that generates both PE and PC. *EPT1* was overexpressed, which resulted in increases in both PE and PC synthesis. Moreover, the *EPT1* overexpression strain is hypervirulent in mice and causes them to succumb to system infection more rapidly than wild-type. In contrast, disruption of *EPT1* causes loss of PE and PC synthesis by the Kennedy pathway, and decreased kidney fungal burden during the mouse systemic infection model, indicating a mild loss of virulence. In addition, the *ept1*Δ*/*Δ mutant exhibits decreased cytotoxicity against oral epithelial cells *in vitro*, whereas the *EPT1* overexpression strain exhibits increased cytotoxicity. Taken altogether, our data indicate that mutations that result in increased PE synthesis cause greater virulence and mutations that decrease PE synthesis attenuate virulence.

## Introduction

*Candida albicans* is a fungus that typically resides as a commensal in the gastrointestinal tract of up to 70% of healthy individuals, as well as within the oral mucosa (Bouza and Munoz, 2008). However, *C. albicans* can cause vaginal infections as well as opportunistic oral and systemic infections, which are more commonly seen in immunocompromised or immunosuppressed individuals (Kabir et al., 2012). Systemic blood stream infections (BSIs) are of particular concern as they have mortality rates of approximately 30–50% (Williams and Lewis, 2011), and *Candida* spp. are the fourth most common causes of BSIs in the United States (Kabir et al., 2012). Treatment of systemic infections has encountered some limitations as a result of poor oral availability and high drug toxicity of some drugs (Letscher-Bru and Herbrecht, 2003; Mukherjee et al., 2005). In addition, resistance to standard antifungal treatments such as fluconazole, by *Candida* spp., is an emerging issue (Sanguinetti et al., 2015). It is therefore imperative that novel drug targets are discovered.

Phospholipid biosynthetic pathways are an attractive area to search for drug targets, as phospholipids are the major structural lipids that form cellular membranes (de Kroon et al., 2013). Cells must therefore synthesize them from precursors acquired from their environment to support growth during infection. In addition to having a structural role within the cell, intermediate phospholipid metabolites can act as second messengers, and may therefore serve important regulatory functions (Exton, 1994; de Kroon et al., 2013). As such, they are required for the growth and pathogenesis of *C. albicans*.

**FIGURE 1:**
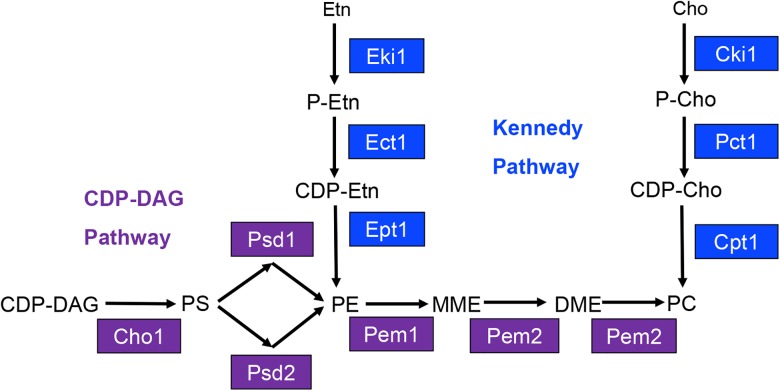
Model for yeast phosphatidylserine (PS), phosphatidylethanolamine (PE), and phosphatidylcholine (PC) biosynthesis. Proteins involved in CDP-DAG pathway mediated phospholipid biosynthesis are shown in purple, and those involved in Kennedy pathway mediated phospholipid biosynthesis are shown in blue. CDP-DAG, cytidine diphosphate diacylglycerol; MME, monomethyl phosphatidylethanolamine; DME, dimethyl-phosphatidylethanolamine; Etn, ethanolamine; Cho, choline; P-Etn, phosphoethanolamine; P-Cho, phosphocholine; CDP-Etn, cytidinediphosphoethanolamine; CDP-Cho, cytidinediphosphocholine.

The most abundant phospholipids in *C. albicans* are phosphatidylglycerol (PG), cardiolipin (CL), phosphatidylserine (PS), phosphatidylethanolamine (PE), phosphatidylcholine (PC), and phosphatidylinositol (PI) (Singh et al., 2010; Cassilly et al., 2017). The major biosynthetic pathway for the phospholipids PS, PE, and PC in *C. albicans* is known as the cytidine-diphosphate diacylglycerol (CDP-DAG) pathway (Figure 1) (Henry et al., 2012). Within this pathway, the common phospholipid precursor CDP-DAG is condensed with serine to form PS by the PS synthase Cho1 (Bae-Lee and Carman, 1984; Henry et al., 2012). PS is then decarboxylated by one of two PS decarboxylases, Psd1 or Psd2, to form PE (Henry et al., 2012). Based on work from *S. cerevisiae*, PE is predicted to be sequentially methylated by the PE methyltransferases Pem1 and Pem2 to form PC (Henry et al., 2012).

Previously, our lab has demonstrated that the *cho1*Δ*/*Δ and *psd1*Δ*/*Δ *psd2*Δ*/*Δ mutants are avirulent in the mouse model of systemic infection (Chen et al., 2010). Interestingly, these mutants are avirulent despite having the ability to utilize an alternative phospholipid biosynthetic pathway known as the Kennedy pathway. This pathway uses exogenous ethanolamine or choline to synthesize PE or PC, respectively (Figure 1) (Gibellini and Smith, 2010). We have recently reported that the Kennedy pathway alone cannot support virulence because the *cho1*Δ*/*Δ and *psd1*Δ*/*Δ *psd2*Δ*/*Δ mutants cannot import sufficient ethanolamine from the host to support PE synthesis (Davis et al., 2018). Growth of either mutant is restored *in vitro* in medium containing >100 μM ethanolamine, which is higher than the estimated concentration of ∼30 μM within the host (Houweling et al., 1992; Davis et al., 2018). In addition, virulence is restored if PE synthesis is supported by heterologous expression of the *Arabidopsis thaliana* serine decarboxylase (AtSDC) (Davis et al., 2018). This enzyme allows for decarboxylation of cytoplasmic serine to yield ethanolamine, which can be used to synthesize PE via the Kennedy pathway. Thus, AtSDC bypasses deficiencies due to importing low levels of exogenous ethanolamine (Davis et al., 2018). Therefore, the wild-type fungus requires the CDP-DAG pathway to generate enough PE to cause disease since it cannot import enough ethanolamine to compensate for PE loss using the Kennedy pathway. Loss of PE synthesis should also impact downstream PC synthesis, and the impact of PC on virulence has been largely unstudied in this organism. In this study, we sought to determine if the loss of PC biosynthesis from the CDP-DAG pathway would attenuate virulence, or if the Kennedy pathway could compensate for this loss. In addition, we directly determined if Kennedy pathway mediated production of PE and/or PC is also important for virulence in *C. albicans* when the CDP-DAG pathway is still functional.

## Materials and Methods

### Strains and Growth Media

*Candida albicans* strains used in this study were derived from the SC5314 background (Gillum et al., 1984) (Table 1). Standard medium used for culturing *C. albicans* was YPD (1% yeast extract, 2% dextrose, and 2% peptone) and cultures were maintained aerobically at 30°C in a shaking incubator at 225 RPM (Styles, 2002). DH5-α *Escherichia coli* (NEB, C2987I) were used for plasmid construction (Table 2), and were cultured in LB broth (1% tryptone, 1% NaCl, 0.5% yeast extract) in a rotating incubator at 37°C (Guthrie and Fink, 2002).

**Table 1:** Strains used in this study.

Strain	Parent strain	Genotype	Source
SC5314	Clinical isolate	Prototrophic wild type	Gillum et al., 1984
YLC394	SC5314	*PEM1/pem1Δ-SAT1*	This Study
YLC401	YLC394	*PEM1/pem1Δ-FRT*	This Study
YLC417	YLC401	*pem1Δ/pem1::P_MET3_-PEM1-SAT1*	This Study
YLC393	SC5314	*PEM2/pem2Δ-SAT1*	This Study
YLC400	YLC393	*PEM2/pem2Δ-FRT*	This Study
YLC405	YLC400	*pem2Δ/Δ-SAT1*	This Study
YLC406	YLC405	*pem2Δ/Δ-FRT*	This Study
BTY38	YLC406	*PEM1/pem1Δ pem2Δ/Δ-SAT1*	This Study
BTY66	BTY38	*PEM1/pem1Δ pem2Δ/Δ-FRT*	This Study
BTY72	BTY66	*pem1Δ/Δ pem2Δ/Δ-SAT1*	This Study
BTY77	BTY72	*pem1Δ/Δ pem2Δ/Δ-FRT*	This Study
BTY138	BTY77	*pem1*Δ*/*Δ *pem2*Δ*/pem2*Δ*::PEM2-SAT1*	This Study
BTY147	BTY138	*pem1*Δ*/*Δ *pem2*Δ*/pem2*Δ*::PEM2 FRT*	This Study
BTY167	BTY147	*pem1*Δ*/*Δ*::PEM1 pem2*Δ*/pem2*Δ*::PEM2-SAT1*	This Study
BTY169	BTY167	*pem1*Δ*/*Δ*::PEM1 pem2*Δ*/pem2*Δ*::PEM2-FRT*	This Study
BTY88	SC5314	*P_ENO1_::P_ENO1_-EPT1*	This Study
BTY97	SC5314	*EPT1/ept1*Δ*-SAT1*	This Study
BTY101	BTY97	*EPT1/ept1*Δ*-FRT*	This Study
BTY104	BTY101	*ept1*Δ*/*Δ*-SAT1*	This Study
BTY108	BTY104	*ept1*Δ*/*Δ*-FRT*	This Study
BTY130	BTY108	*ept1*Δ*/*Δ *P_ENO1_::P_ENO1_-EPT1-SAT1*	This Study
BTY192	BTY108	*ept1*Δ*/*Δ*::EPT1-SAT1*	This Study
BTY200	BTY192	*ept1*Δ*/*Δ*::EPT1-FRT*	This Study


**Table 2:** Plasmids used in this study.

Plasmid	Description	Parent vector
pSFS2A	*SAT1* flipper cassette; (CAT^R^)	Reuss et al., 2004
pYLC390	*PEM1* knockout construct; 5′ NCR (KpnI-ApaI) + 3′ NCR (Sac II-I) of *PEM1*	pSFS2A
pYLC388	*PEM2* knockout construct; 5′ NCR (KpnI-ApaI) + 3′ NCR (Sac II-I) of *PEM2*	pSFS2A
pYLC314	Methionine repressible expression cassette; *SAT1-P_MET3_* (EcoRI-EcoRI); (AMP^R^)	pBluescript SK+
pYLC315	Methionine repressible expression cassette; *SAT1-P_MET3_* (EcoRI-EcoRI); (AMP^R^)	pBluescript SK+
pYLC410	*PEM1* repressible expression vector; 5′ NCR of *PEM1* (KpnI-ApaI) + *SAT1-P_MET3_* (EcoRI-EcoRI)	pYLC315
pYLC414	*PEM1* repressible expression vector; 5′ NCR of *PEM1* (KpnI-ApaI) + *SAT1-P_MET3_* (EcoRI-EcoRI) + *PEM*1 ORF (SacII-SacI)	pYLC410
pBT33	*PEM2* reintegration construct; 5′ NCR and *PEM2* ORF (KpnI-ApaI) + 3′ NCR (SacII-SacI) of *PEM2*	pYLC388
pBT47	*PEM1* reintegration construct; 5′ NCR + *PEM1* ORF (ApaI-XhoI)	pSFS2A
pBT49	*PEM1* reintegration construct; 5′ NCR and *PEM1* ORF (ApaI-XhoI) + 3′ NCR (Not1-SacI) of *PEM1*	pBT47
pBT1	Overexpression Vector; *PENO1* (BamHI-Not1)	pYLC314
pBT11	*EPT1* overexpression cassette; *PENO1-EPT1* (NotI-SacI)	pBT1
pBT16	*EPT1* knockout construct; 5′ NCR and *EPT1* ORF (KpnI, XhoI) + 3′ NCR (NotI, SacI) of *EPT1* (CAT^R^)	pSFS2A
pBT51	*EPT1* reintegration construct; 5′ NCR + *EPT1* ORF (KpnI-XhoI)	pSFS2A
pBT53	*EPT1* reintegration construct; 5′ NCR + *EPT1* ORF (KpnI-XhoI) + 3′ NCR (SacII-SacI)	pBT51


### Plasmid Construction

Primers described in this section are shown in Supplementary Table S1. The *PEM1* knockout vector was created using the *SAT1* flipper plasmid pSFS2A (Cat^R^) (Reuss et al., 2004). A 589 bp region of the *PEM1* (*CHO2/CR_02540W_A/orf19.169*) 5′ non-coding region (NCR) was amplified using the primers JCO197 and JCO198 which introduced *KnpI* and *ApaI* sites, respectively. This fragment was cloned into the *KpnI* and *ApaI* sites of pSFS2A. In addition, a 589 bp 3′ NCR of *PEM1* was amplified using JCO199 and JCO200, and was subsequently cloned into the *SacII* and *SacI* sites of pSFS2A to create the pYLC390 *PEM1* knockout vector.

In addition to the *PEM1* knockout vector, a *P_MET3_* repressible *PEM1* expression vector was also created. To create this vector JCO165 and JCO166 were used to amplify an *EcoRI*-flanked *SAT1*-*P_MET3_* fragment from pYLC229 (Chen et al., 2008), which was subsequently cloned into the *EcoRI* site of a pBluescript SK+ plasmid to yield two isogenic clones: pYLC314 and pYLC315. The 5′ NCR of *PEM1* was amplified from genomic *C. albicans* DNA with JCO199 and JCO200 as a *KpnI-ApaI* fragment and cloned into pYLC315 (*KpnI-ApaI*) upstream of the *SAT1* marked *P_MET3_* promoter yielding pYLC410. To complete the construct, the *PEM1* ORF was amplified along with the 3′NCR with JCO215 and JCO216, and this *SacII-SacI* fragment was cloned into pYLC410 downstream of the *P_MET3_* promoter (*SacII-SacI*) to yield the *PEM1* conditional expression vector pYLC414.

The *SAT1* flipper vector pSFS2A was used to create a *PEM2* knockout vector, pYLC388 (Reuss et al., 2004). A 592 bp 5′ NCR to *PEM2* (*OPI3*/C3_06570C_A/orf19.7446) was amplified using JCO192 and JCO193 and cloned in the *KpnI* and *ApaI* sites of pSFS2A. A 587 bp NCR 3′ to the *PEM2* ORF was amplified using JCO194 and JCO195 and cloned into the *SacII* and *SacI* sites, resulting in pYLC388.

pYLC388 was used to create a reintegration construct for *PEM2*. The 5′ NCR was released from pYLC388 by restriction with *ApaI* and *KpnI*. A 1,297 bp fragment including the *PEM2* ORF (*OPI3*/C3_06570C_A/orf19.7446) and approximately 500 bp of NCR 5′ to the ORF was amplified with BTO67 and JCO192. This fragment was subcloned into pYLC388 at the *ApaI-KpnI* sites to create pBT33.

The *PEM1* reintegration construct was created using pSFS2A (Reuss et al., 2004). pSFS2A was linearized using ApaI and XhoI. A 3451 bp fragment including approximately 500 bp of the 5′ NCR of *PEM1*, the *PEM1* ORF (CHO2/CR_02540W_A/orf19.169), and approximately 300 bp of the 3′ NCR of *PEM1* was amplified using BTO52 and BTO53 to introduce *ApaI-XhoI* sites, and subsequently was cloned into the linearized vector to create pBT47. pBT47 was linearized using *NotI* and *SacI*. A 622 bp NCR 3′ to the *PEM1* ORF (-356 to -978) was amplified using BTO54 and BTO55 to introduce *NotI-SacI* sites, and subsequently cloned into the linearized pBT47 vector to create the *PEM1* reintegration vector, pBT49.

For the construction of an overexpression vector, pYLC314 was used. The *P_MET3_* promoter was removed from pYLC314 by restriction with *PstI*, which flanks both sides of the insert. The constitutively active *P_ENO1_* promoter, +928 to +1 5′ of the *ENO1* ORF (C1_08500C_A/orf19.395), was amplified from wild type *C. albicans* genomic DNA using the primers BTO30 and BTO31 to introduce *BamHI* and *NotI* sites, and was cloned into the *BamHI* and *NotI* sites of pYLC314 to create pBT1.

To overexpress *EPT1*, a 1,509 bp fragment including the *EPT1* ORF (C7_02690C_A/orf19.3695) and approximately 300 bp of NCR 3′ to the ORF was amplified from *C. albicans* genomic DNA using BTO35 and BTO36, and was cloned into the *NotI* and *SacI* sites of pBT1 to create the *EPT1* overexpression vector, pBT11.

To create an *EPT1* knockout vector, the 5′ and 3′ NCRs of the *EPT1* ORF (C7_02690C_A/orf19.3695) were cloned into pSFS2A plasmid flanking the *SAT1* flipper construct (Reuss et al., 2004). First, a 471 bp fragment of the 5′ NCR of the *EPT1* ORF was amplified from *C. albicans* genomic DNA using BTO44 and BTO45 and subsequently cloned into the *KpnI* and *XhoI* sites of pSFS2A. In addition, a 494 bp fragment of the 3′ NCR of *EPT1* was amplified with BTO46 and BTO47 and cloned into the *NotI* and *SacI* sites to create pBT16.

To create an *EPT1* reintegration construct a 2312 bp fragment including the *EPT1* ORF, 903 bp 5′ NCR of the *EPT1* ORF, and 200 bp of the 3′ NCR was amplified from genomic *C. albicans* DNA using TRO1044 and TRO1045. This PCR product was cloned into pSFS2A at the *KpnI* and *Xho*I sites to create pBT51. A 596 bp fragment of the 3′ NCR (+202 to +798) was amplified from genomic DNA using TRO1046 and TRO1047 and cloned into the *SacI* and *SacII* sites of pBT51 to create the finalized *EPT1* reintegration construct, pBT53.

### *Candida albicans* Strain Construction

Plasmids were cut at the indicated restriction site(s) and linearized fragments were purified using the QIAquick PCR Purification Kit (QIAGEN, 28106) for single restriction digests, or the QIAquick Gel Extraction Kit (QIAGEN, 28704) if restriction digests resulted in multiple fragments. All *Candida albicans* transformations were performed via electroporation as described previously (Hasim et al., 2014) and plates containing 200 μg/mL nourseothricin (GoldBio, N-500-1) were used for selection of *C. albicans* transformants (Table 1). Following transformation, mutations were confirmed via PCR using the indicated primers (Supplementary Table S1).

To create the *pem2*Δ*/*Δ strain, pYLC388 was linearized (*KpnI, SacI*) and transformed into wild type *C. albicans* (SC5314). The *SAT1* marker was removed by *FLP*-mediated recombination from YLC393 using the *SAT1* flipper method to create YLC400 (*PEM2/pem2*Δ) (Reuss et al., 2004). Linearized pYLC388 was again used to transform YLC400, which resulted in the *pem2*Δ/Δ*-SAT1* strain (YLC405). The *SAT1* marker was removed by recombination from YLC405 to create YLC406 (*pem2*Δ*/*Δ*)*.

YLC406 was transformed with a linearized pYLC390 fragment (*KpnI, SacI*) to create the *PEM1/pem1*Δ*-SAT1 pem2*Δ*/*Δ mutant BTY38. The *SAT1* marker was removed by recombination to create BTY66 (*PEM1/pem1*Δ *pem2*Δ*/*Δ). BTY66 was again transformed with linearized pYLC390 (*KpnI, SacI*) to create a *pem1*Δ*/*Δ*-SAT1 pem2*Δ*/*Δ mutant (BTY72), and the *SAT1* marker was removed by recombination to create BTY77 (*pem1*Δ*/*Δ *pem2*Δ*/*Δ).

To reintegrate *PEM1* and *PEM2* into the homozygous double mutant, pBT33 was linearized (*KpnI, SacI*) and transformed into BTY77 to create a *pem1*Δ*/*Δ *pem2*Δ*/pem2*Δ*::PEM2-SAT1* strain, BTY138. The *SAT1* marker was removed by recombination from BTY138, resulting in BTY147 (*pem1*Δ*/*Δ *pem2*Δ*/pem2*Δ*::PEM2*). BTY147 was transformed with linearized pBT49 (*ApaI, SacI*) to create BTY167 (*pem1*Δ*/*Δ*::PEM1 pem2*Δ*/pem2*Δ*::PEM2-SAT1*). The *SAT1* marker was removed by recombination from BTY167 to create the *pem1*Δ*/*Δ*::PEM1 pem2*Δ*/pem2*Δ*::PEM2* mutant (BTY169).

To create a repressible *PEM1* mutant, SC5314 was transformed with linearized pYLC390 (*KpnI-SacI*) to yield a *PEM1/pem1*Δ*-SAT1* strain (YLC394). The *SAT1* marker was removed by recombination from YLC394 to yield a *PEM1/pem1*Δ strain (YLC401). YLC401 was then transformed with the *PEM1* conditional expression vector pYLC414 (*KpnI*) to yield a *pem1*Δ*/pem1*Δ*::P_MET3_-PEM1-SAT1* strain (YLC417).

To overexpress *EPT1*, SC5314 was transformed using linearized pBT11 (MscI) to create BTY88 (*P_ENO1_::P_ENO1_-EPT1*). To knock out *EPT1*, pBT16 was linearized (*KpnI, SacI*) and transformed into SC5314 resulting in an *EPT1/ept1*Δ*-SAT1* strain (BTY97). The *SAT1* marker was removed by recombination from BTY97, resulting in BTY101 (*EPT1/ept1*Δ). BTY101 was transformed with linearized pBT16 again to create the *ept1*Δ*/*Δ*-SAT1* strain, BTY104. The *SAT1* marker was removed by recombination from BTY104 to create BTY108 (*ept1*Δ*/*Δ), so that a reintegrant strain could be produced. Two distinct types of reintegrant were produced: one in which the *EPT1* gene was constitutively expressed, and another in which one allele of *EPT1* was reintegrated into the *EPT1* locus under its native promoter. For the first reintegrant, the overexpression cassette pBT11 was linearized (*MscI*) and transformed into BTY108, resulting in the *ept1*Δ/Δ *P_ENO1_::P_ENO1_-EPT1* strain (BTY130). To reintegrate the gene under its native promoter and at the *EPT1* locus, pBT53 was linearized using *KpnI* and *SacI* and subsequently transformed into BTY108 to yield BTY192 (*ept1*Δ*/*Δ*::EPT1-SAT1*). The *SAT1* marker was then removed by recombination to yield BTY200 (*ept1*Δ*/*Δ*::EPT1*).

### Growth Curves

To determine choline auxotrophy, strains were cultured overnight in liquid YNB medium [0.67% yeast nitrogen base containing ammonium sulfate (BD Difco, 291940), 2% dextrose], and then diluted to OD_600__nm_ = 0.1 in triplicate in fresh YNB (Styles, 2002). Growth was measured at OD_600__nm_ every 2 h for 12 h or at other time points as indicated. To rescue choline auxotrophy YNB medium was supplemented with choline chloride (Fisher Scientific, O1972-250), glycerophosphocholine (Sigma-Aldrich, G5291-10MG), or lysophosphatidylcholine (Avanti Polar Lipids INC., 845875P).

### Lipid Extraction and TLC

*Candida albicans* strains were cultured overnight in 5 mL YNB medium supplemented with 0.25 mM L-methionine/L-cysteine (represses *PEM1* in pYLC417), and then diluted to an OD_600__nm_ = 0.4 in 25 mL of YNB with 0.25 mM L-methionine/L-cysteine or YNB supplemented with 1 mM choline chloride and 0.25 mM L-methionine/L-cysteine. Cultures were maintained at 30°C for 12 h at 225 RPM and then transferred to 50 mL conical tubes and pelleted at 3000 RPM for 10 min. Pellets were lyophilized and weighed to normalize for the number of cells present. Following lyophilization, pellets were washed with 25 mL water and resuspended in 3 mL ethanol:water (4:1). Suspensions were heated in boiling water for 15 min and pelleted at 3,000 RPM for 10 min. The supernatant was transferred to new tubes and back extractions were completed twice using 1 mL of ethanol:water (4:1) following the above procedure. Lipids were dried under nitrogen gas and resuspended in chloroform:methanol (2:1). TLC plates were washed once with chloroform:methanol (2:1) and dried at 100°C for 15 min. Lipid extracts were spot inoculated on TLC plates (Millipore, HX377581) and chloroform:ethanol:water:triethylamine (35:30:7:35) was used as a solvent system for separation (Chen et al., 2010). Phospholipids were visualized under UV following treatment with primuline (Fisher Scientific, 8064-60-6).

### Mouse Models

Outbred male ICR mice were obtained from Envigo for use in this study. *C. albicans* strains were cultured overnight in 50 mL YPD at 30°C and 225 RPM. After approximately 16 h they were transferred to 50 mL Falcon tubes and centrifuged at 3,500 RPM for 5 min. The pellets were subsequently washed twice with 25 mL of water. Cells were counted via hemocytometer and diluted to 5 × 10^6^ cells/mL unless otherwise noted. Mice were injected via the lateral tail vein with 0.1 mL of the *C. albicans* suspension. *C. albicans* suspensions were plated on YPD and incubated overnight at 30°C to determine cell viability. Following infection, mice were monitored for signs of illness for 21 days and were sacrificed after succumbing to infection.

For experiments in which fungal burden was measured, mice were sacrificed 5 days post infection and kidneys were harvested. Kidneys were placed in 1 mL water within pre-weighed whirl-pack bags and homogenized. Serial dilutions (10^-1^, 10^-2^, and 10^-3^) were prepared in water, and 1 mL of each dilution was added to 1.1% Noble agar pours (3.5 mL) at 55°C and poured onto YPD plates in duplicate for each dilution set. Plates were incubated at 30°C overnight and colony forming unit (CFUs) were counted after 24 h.

### Ethics Statement

All mouse model experiments in this study were performed under an animal protocol (0016-0714) that was approved by the University of Tennessee Institutional Animal Care and Use Committee (IACUC). We followed the ethical guidelines set forth by the National Institute of Health (NIH) for the ethical treatment of animals.

### *In vitro* Cytotoxicity Assays

The FaDu oral epithelial cell line was used in this study (Martinez-Lopez et al., 2006). Cells were routinely cultured in EBSS medium (Fisher Scientific, SH30024.01) with 10% fetal bovine serum (Fisher Scientific, BP1600-100) and 1% penicillin/streptomycin (Invitrogen, 15140-122) at 37°C and 5% CO_2_. Prior to the cell killing assay, 5 × 10^5^ cells/mL of FaDu cells were plated in a 24 well plate. For each *C. albicans* strain, 1 mL of FaDu cells was plated in triplicate. *C. albicans* strains were cultured overnight in 5 mL YPD, and then washed twice with sterile water. Cells were resuspended in 10 mL H_2_O and counted via hemocytometer. Solutions containing 2.5 × 10^6^ cells/mL of each strain (MOI = 5) were made in EBSS supplemented with 2% Human Serum (MP Biomedicals, ICN2930149) and 1% penicillin/streptomycin.

Wells containing FaDu cells were aspirated and 1 mL of *C. albicans* cell suspensions were added to FaDu cells for co-incubation. In addition, the following control wells were included in triplicate: *C. albicans* alone, FaDu cells alone, medium alone, FaDu cells with lysis solution, and lysis solution with medium. Plates were centrifuged at 250 ×*g* for 5 min and incubated for 4 h at 37°C at 5% CO_2_. Following incubation, plates were spun at 250 ×*g* for 5 min, and 50 μL of each supernatant were assayed for LDH release using the CytoTox 96^®^ Non-Radioactive Cytotoxicity Assay (Promega, G1780) using the manufacturer’s protocol. Cytotoxicity was expressed as a relative percentage of the average wild type cytotoxicity, and three biological replicates were performed (each with technical replicates in triplicate).

### PC Synthesis Assay

This procedure was done as previously described for a phosphatidylserine synthase assay with some changes (Bae-Lee and Carman, 1984; Cassilly et al., 2017). The optimal PC synthesis assay mixture contained 50 mM Tris-HCl pH 7.5, 0.1% Triton X-100, 0.5 mM MnCl_2_ and 0.1 mM DAG (Avanti Polar Lipids INC.) added as a suspension in 1% Triton X-100 and 0.5 mg protein in a total volume of 0.1 mL. The assay was performed by monitoring the incorporation of 0.5 mM cytidine diphosphocholine (Sigma-Aldrich) spiked with 10% by volume ^[14C]^-methyl cytidine diphosphocholine (∼0.2 μCi) into the chloroform-soluble product at 37°C for a predetermined amount of time. The reaction was terminated by the addition of 1 mL chloroform:methanol (2:1). Following this the reaction was extracted as previously described. Samples were fully dried in scintillation vials, 2.5 mL scintillation fluid was added to each vial, and they were measured with a liquid scintillation counter (Tri-Carb 2900TR).

### *In vivo* PE Synthesis Assay

Cultures were grown to logarithmic phase at 30°C in YNB in media supplemented with 100 μM choline chloride and 0.2 μCi/mL of ^[14C]^ethanolamine. Phospholipids were isolated as described in Surlow et al. (2014). Lipid extracts representing equivalent amounts of optical density units (ODUs) were spotted onto silica gel TLC plates and plates were developed in chloroform:ethanol:water:triethylamine (30:35:7:35). A Typhoon 8600 phosphorimager was used for visualization and quantitation of PC and PE regions of intensity (ROI).

### Statistical Analyses and BLAST Parameters

All statistical analyses were carried out using GraphPad Prism (version 7.04). For mouse survival curves the log-rank (Mantel–Cox) test was performed and for all other analyses unpaired *t*-tests between the indicated strains were used to determine statistical significance. For all analyses, *p* < 0.05 was used as a cutoff to determine statistical significance. To align protein sequences from *S. cerevisiae* to *C. albicans*, Basic Local Alignment Search Tool Protein–Protein (BLASTP) was used (NCBI, CGD). Sequences were obtained for *S. cerevisiae* S288C from the Saccharomyces Genome Database (SGD) and BLASTP was performed via the Candida Genome Database (CGD). The default settings were used to query assembly 22 of *C. albicans* SC5314 for hits. These settings included allowgaps = yes, gapext = 1, gapopen = 11, matrix = BLOSUM62.

## Results

### Disruption of *PEM1* and *PEM2* Causes Choline Auxotrophy in *C. albicans*

**FIGURE 2:**
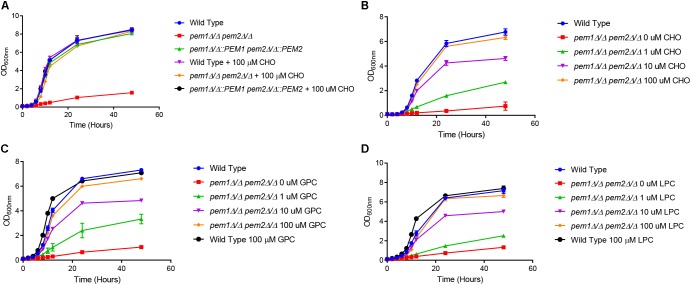
The *pem1*Δ*/*Δ *pem2*Δ*/*Δ mutant is a choline auxotroph. **(A)** Wild type, *pem1*Δ*/*Δ *pem2*Δ*/*Δ, and *pem1*Δ*/*Δ*::PEM1 pem2*Δ*/*Δ*::PEM2* strains were grown overnight in minimal media, diluted to an OD_600nm_ = 0.1, and then cultured for 48 h in minimal media, with or without 100 μM choline (CHO). Wild type and *pem1*Δ*/*Δ *pem2*Δ*/*Δ were also cultured for 48 h in minimal media, with or without varying concentrations of **(B)** choline (CHO), **(C)** glycerophosphocholine (GPC), or **(D)** lysophosphatidylcholine (LPC). These assays were performed with three biological replicates.

To determine if CDP-DAG mediated PC biosynthesis is required for virulence in *C. albicans*, methylation of PE to PC was disrupted by constructing a *pem1*Δ*/*Δ *pem2*Δ*/*Δ knockout mutant using the *SAT1* flipper method (Reuss et al., 2004). The *pem1*Δ*/*Δ *pem2*Δ*/*Δ mutant is expected to be a choline auxotroph because it can only make PC from imported choline via the Kennedy pathway (Figure 1). Therefore, growth of the *pem1*Δ*/*Δ *pem2*Δ*/*Δ mutant was observed for 48 h in minimal media (YNB) with or without 100 μM choline supplementation (Figure 2A). Little growth occurred in minimal medium lacking choline, indicating that the *pem1*Δ*/*Δ *pem2*Δ*/*Δ mutant cannot synthesize PC efficiently due to disruption of the CDP-DAG pathway. Residual growth of *pem1*Δ*/*Δ *pem2*Δ*/*Δ may be due to the ability to store PC or choline within the cell. Addition of 100 μM choline or reintegration of the methyltransferase genes (*pem1*Δ*/*Δ*::PEM1 pem2*Δ*/*Δ*::PEM2* strain) restored growth to wild-type levels, which indicates that exogenous choline can be used to synthesize PC via the Kennedy pathway. In fact, choline levels as low as 10 μM also restored growth to nearly wild-type levels (Figure 2B). Choline is found at ∼11 μM in rodent serum (Klein et al., 1993).

To further investigate the ability of *C. albicans* to acquire choline from other exogenous sources we tested several choline-containing compounds that are commonly found in human and rodent tissues/serum, including lysophosphatidylcholine (LPC) and glycerophosphocholine (GPC), (Croset et al., 2000; Ilcol et al., 2005) to determine if they could also restore growth. LPC is as high as 450 μM in rat serum (Suarez-Garcia et al., 2017) and GPC is as high as 4.5 μM, but GPC can be over 1 mM in homogenized brain tissue and approximately 270 μM/g protein in homogenized kidney tissue (Bauernschmitt and Kinne, 1993; Klein et al., 1993). The growth of the *pem1*Δ*/*Δ *pem2*Δ*/*Δ mutant was restored to the wild type level in minimal media when supplemented with either 100 μM GPC or LPC, and was restored to nearly wild-type levels even at 10 μM (Figure 2C,D).

To confirm that the *pem1*Δ*/*Δ *pem2*Δ*/*Δ mutant’s lack of growth in choline-free media correlates with loss of PC synthesis, total lipids were extracted from cells growing in minimal medium lacking a source of choline, and analyzed by thin layer chromatography (TLC) (Supplementary Figure S1). While wild type *C. albicans* was able to produce PC regardless of supplementation, the *pem1*Δ*/*Δ *pem2*Δ*/*Δ mutant failed to produce PC unless supplemented with choline.

Single mutants for both *PEM1* and *PEM2* were also tested for PC synthesis. For *PEM1*, a *pem1*Δ*/PEM1::P_MET3_-PEM1* conditional knockout was used, and for *PEM2* a *pem2*Δ*/*Δ conventional knockout was made. Both were cultured in YNB with or without 1 mM choline (Supplementary Figure S1). In addition, the YNB media included 0.25 mM cysteine/methionine to shut off expression of *PEM1* in the *pem1*Δ*/PEM1::P_MET3_-PEM1* strain. The *pem1*Δ*/PEM1::P_MET3_-PEM1* strain was fully capable of PC production even in the absence of choline when methionine/cysteine were present, but the *pem2*Δ*/*Δ mutant failed to produce PC unless supplemented with choline and there was an increase in the intermediate product monomethylethanolamine. This resembles *S. cerevisiae*, in which *PEM2* has overlapping substrate specificity and can methylate PE (Preitschopf et al., 1993; Henry et al., 2012). This also indicates that Pem1 cannot efficiently methylate the intermediate products monomethylethanolamine or dimethylethanolamine to PC on its own.

### PE From the CDP-DAG Pathway Accumulates in the *pem1*Δ*/*Δ *pem2*Δ*/*Δ Mutant

**FIGURE 3:**
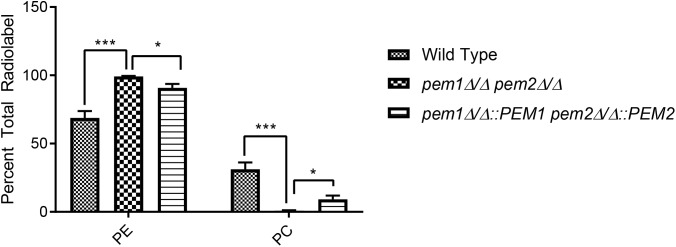
The *pem1*Δ*/*Δ *pem2*Δ*/*Δ mutant cannot produce PC via the CDP-DAG pathway. Strains were cultured in the presence of ^[14C]^ethanolamine and incorporation of the radiolabel into PE and PC was quantified using thin-layer chromatography (TLC) of extracted lipids (^∗∗∗^*p* < 0.001, ^∗^*p* < 0.05; unpaired *t*-test compared to wild-type). This assay was performed with biological replicates in triplicate.

If PE methylation to PC is blocked, it is expected that PE will accumulate in the cell and PC from this pathway will decrease. To test this, ^[14C]^ethanolamine radiolabeling assays of whole cells were performed using the *pem1*Δ*/*Δ *pem2*Δ*/*Δ mutant. This experiment measures the accumulation of PE that is derived from the Kennedy pathway. There was an approximate 1.44-fold increase in radiolabeled PE in *pem1*Δ*/*Δ *pem2*Δ*/*Δ mutants as compared to the wild type (^∗∗∗^*p* < 0.001) (Figure 3) and little or no PC was detected. Thus, blockage of PE methylation results in the expected depletion of labeled PC and a buildup of labeled PE. When the methyltransferases are reintegrated into the mutant, statistically significant decreases in PE radiolabel accumulation and concurrent increases in PC radiolabel accumulation are measured relative to the mutant (^∗^*p* < 0.05). These data do not measure the total PE and PC in the cells, as the phospholipid pools produced by the CDP-DAG pathway for PE or the Kennedy pathway for PC are unlabeled (Figure 1, 3), but the data do reveal that blockage of the CDP-DAG pathway at this point increases PE and decreases PC specifically generated from imported ethanolamine.

### Blockage of PC Synthesis by the PE Methyltransferase Mutant *pem1*Δ*/*Δ *pem2*Δ*/*Δ Results in Hypervirulence

**FIGURE 4:**
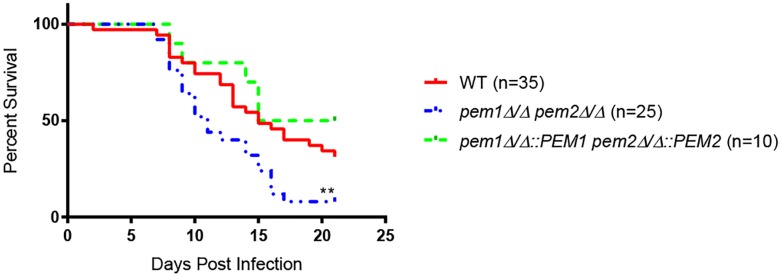
The *pem1*Δ*/*Δ *pem2*Δ*/*Δ mutant is hypervirulent. Mice were infected via the lateral tail vein with 3 × 10^5^ wild type (WT), *pem1*Δ*/*Δ *pem2*Δ*/*Δ, or *pem1*Δ*/*Δ*::PEM1 pem2*Δ*/*Δ*::PEM2* cells. Mice infected with the *pem1*Δ*/*Δ *pem2*Δ*/*Δ mutant succumb to infection more rapidly than those infected with wild type *C. albicans* [^∗∗^*p* < 0.01 compared to wild-type; log-rank (Mantel–Cox) test]. The number of mice representing each strain is shown in parenthesis beside each strain.

Choline, GPC, and LPC can fully support growth of *pem1*Δ*/*Δ *pem2*Δ*/*Δ mutants *in vitro* (Figure 2). These compounds are all found in the host, and therefore can be used to make PC by pathways that are alternatives to the CDP-DAG pathway, such as the Kennedy pathway (Figure 1). However, it was still possible that loss of *PEM1* and *PEM2* would affect virulence, so the mutants were tested in a mouse model of systemic infection. Outbred ICR mice were infected with 3 × 10^5^ cells each of either wild type (*n* = 35), *pem1*Δ*/*Δ *pem2*Δ*/*Δ (*n* = 25), or *pem1*Δ*/*Δ*::PEM1 pem2*Δ*/*Δ*::PEM2* (*n* = 10) via the tail vein. The *pem1*Δ*/*Δ *pem2*Δ*/*Δ strain is fully virulent (Figure 4), indicating that PC biosynthesis by the CDP-DAG pathway is not required for full virulence, as the Kennedy pathway can synthesize enough PC *in vivo* to support virulence using choline obtained from the host.

Moreover, mice infected with the *pem1*Δ*/*Δ *pem2*Δ*/*Δ mutant succumb to infection more rapidly than those infected with wild type (^∗∗^*p* < 0.01) (Figure 4). This was unexpected, and we hypothesized that the *pem1*Δ*/*Δ *pem2*Δ*/*Δ mutant might be more virulent because the blockage of PC biosynthesis by the CDP-DAG pathway leads to increased PE, as seen *in vitro* (Figure 3). This could be because a block of the CDP-DAG pathway upregulates an alternative PE synthesis pathway, such as the Kennedy pathway. Alternatively, there may just be a buildup of PE as it cannot be methylated. In either case, this may have a downstream effect on virulence. If so, then overexpression of the Kennedy pathway (overexpression of PE synthesis enzymes by an alternative means), should increase virulence in a similar manner.

### In Contrast to *S. cerevisiae*, in *C. albicans* One Enzyme, Ept1, Catalyzes the Final Step for Both PE and PC Biosynthesis in the Kennedy Pathway

**FIGURE 5:**
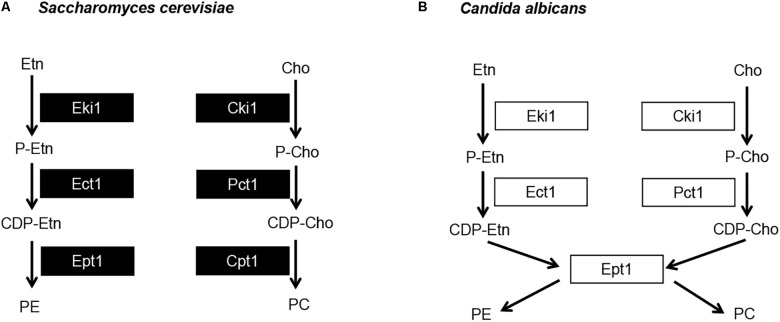
*Candida albicans* has a single dual purpose ethanolamine/cholinephosphotransferase. **(A)** Model for the *S. cerevisiae* Kennedy pathway and **(B)** proposed model for *C. albicans* Kennedy pathway. Etn, ethanolamine; Cho, choline; P-Etn, phosphoethanolamine; P-Cho, phosphocholine; CDP-Etn, cytidinediphosphoethanolamine; CDP-Cho, cytidinediphosphocholine.

The Kennedy pathway can use exogenous choline and ethanolamine from the environment to form PC and PE, respectively, and it also makes PC from internal stores of choline that have been released by phospholipase activity (Dowd et al., 2001; Fernandez-Murray et al., 2009). The current model for the *C. albicans* Kennedy pathway is inferred from that characterized in baker’s yeast (Figure 1). In *S. cerevisiae* there are two branches of the Kennedy pathway; the CDP-ethanolamine and CDP-choline branches (Gibellini and Smith, 2010) (Figure 5A). Each branch catalyzes analogous reactions to convert ethanolamine or choline into PE or PC, respectively. To begin synthesis of either PE or PC, ethanolamine or choline are imported into the cell by the ethanolamine/choline importer Hnm1 (Nikawa et al., 1986, 1990). These precursors are then phosphorylated by either ethanolamine (Eki1) or choline (Cki1) kinase to form phosphoethanolamine (P-Etn) or phosphocholine (P-Cho), respectively (Gibellini and Smith, 2010). P-Etn and P-Cho are used to form the high-energy intermediates CDP-ethanolamine and CDP-choline by the ethanolamine and choline cytidyltransferases, Ect1 and Pct1, respectively (Gibellini and Smith, 2010). Finally, diacylglycerol is condensed with CDP-ethanolamine by Ept1 to form PE, or CDP-choline by Cpt1 to form PC, with the release of CMP in both cases (Gibellini and Smith, 2010).

The two *S. cerevisiae* enzymes that represent each step of the Kennedy pathway (one from each branch, Figure 5A) share high sequence identity. For example, when searching the *C. albicans* protein database in the *Candida* Genome Database (CGD) with the Basic Local Alignment Search Tool (BLAST) using the *S. cerevisiae* Eki1 sequence as a query, hits are given for both the *C. albicans* Eki1 (C7_01320W/orf19.6912) and Cki1 (C3_05300C/orf19.6966) sequences (*e*-values of 6e-7 and 8e-87, respectively). The same is true for *S. cerevisiae* Cki1. Thus, this holds true for the enzymes in the first two steps of the Kennedy pathway. However, we recovered only one protein sequence when we used BLAST to search CGD with the *S. cerevisiae* Ept1 or Cpt1 protein sequences. For either *S. cerevisiae* protein, a single *C. albicans* protein is revealed that is designated as Ept1 (C7_02690C/orf19.3695), (*e*-value of 3e-114 for Ept1 and 7e-111 for Cpt1). We hypothesized that *C. albicans* differs from *S. cerevisiae* in that it uses a single phosphotransferase (*C. albicans* Ept1) to catalyze the addition of both CDP-ethanolamine and CDP-choline to DAG to make PE and PC, respectively (Figure 5B). If so, blocking this step of the pathway would impede Kennedy pathway mediated synthesis of both PE and PC. Alternatively, overexpressing it may increase synthesis of both PE and PC as well.

In order to determine if Ept1 alone catalyzes the final step of both branches of the Kennedy pathway in *C. albicans*, an *ept1*Δ*/*Δ knockout strain was generated using the *SAT1* flipper method (Reuss et al., 2004). In addition, *EPT1* was cloned into an overexpression vector under the control of the strong, constitutive *ENO1* promoter in order to create a *P_ENO1_-EPT1* overexpression plasmid. This *P_ENO1_-EPT1* plasmid was integrated into wild-type *C. albicans*. In addition, reintegrants were made for *ept1*Δ*/*Δ in which one allele of *EPT1* was expressed at its native locus and under control of its native promoter or the *P_ENO1_-EPT1* plasmid was used to reintegrate the gene.

**FIGURE 6:**
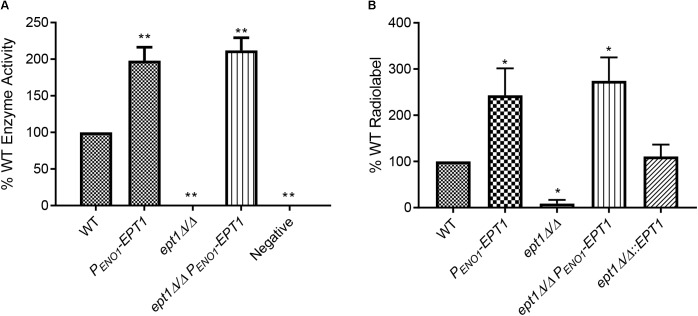
Biosynthesis of both PC and PE via the Kennedy pathway requires *EPT1*. **(A)** Membranes were isolated from all strains and used in an *in vitro* PC synthesis assay, where ^[14C]^CDP-choline incorporation into PC was measured. Enzyme activity was measured as counts per minute per milligram of protein, and is expressed as a percentage of wild-type (^∗∗^*p* < 0.01; unpaired *t*-test compared to wild-type, WT). **(B)** Each strain was cultured in the presence of ^[14C]^ethanolamine and incorporation of the radiolabel into PE was quantified by phosphorimager from thin-layer chromatography (^∗^*p* < 0.05; compared to wild-type, WT, by the unpaired *t*-test). Each of these assays was performed with three biological replicates.

To determine if these alterations in *EPT1* affected PC synthesis from its precursors CDP-choline and diacylglycerol (DAG), as predicted, a CDP-cholinephosphotransferase assay was performed on membranes isolated from each strain. Membranes were isolated from the strains and incubated with ^[14C]^CDP-choline and DAG, and the level of ^[14C]^PC synthesized was measured. Disruption of *EPT1* resulted in a total loss of PC synthesis, while overexpression of *EPT1* in wild-type or *ept1*Δ*/*Δ resulted in twice the level of ^[14C]^PC as found in wild-type (Figure 6A, ^∗∗^*p* < 0.01). Thus, Ept1 is solely responsible for catalyzing PC synthesis from CDP-choline and DAG.

Due to difficulty in obtaining radiolabeled ^[14C]^CDP-ethanolamine, the role for *C. albicans* Ept1 in PE synthesis was tested by measuring synthesis of ^[14C]^PE from ^[14C]^ethanolamine in whole cells. Each strain was cultured in minimal (YNB) media supplemented with ^[14C]^ethanolamine, and lipids were extracted (Surlow et al., 2014), separated by TLC, and quantified to measure incorporation of the radiolabel into PE (Figure 6B, ^∗^*p* < 0.05). Confirming our hypothesis, the *ept1*Δ*/*Δ strain does not incorporate a detectable amount of radiolabel into PE. In contrast, the *P_ENO1_-EPT1* strain incorporates increased radiolabel into PE. Finally, the *ept1*Δ*/*Δ mutant transformed with *P_ENO1_-EPT1* or *EPT1* on its native promoter has a restored ability to incorporate the radiolabel into both phospholipids (Figure 6A,B). Thus, *EPT1* solely catalyzes the final step of both the CDP-choline and CDP-ethanolamine branches of the Kennedy pathway in *C. albicans* (Figure 5B).

### Overexpression of *EPT1* Results in Hypervirulence in the Mouse Systemic Infection Model

**FIGURE 7:**
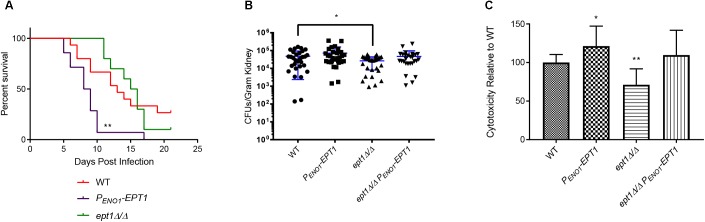
Overexpression of *EPT1* causes hypervirulence. **(A)** Mice infected with *P_ENO1_-EPT1* overexpression strain (*n* = 14) succumb to infection more rapidly than the wild type (WT, *n* = 15) [^∗∗^*p* < 0.01; compared to WT, log-rank (Mantel–Cox) test]. Mice infected with the isogenic *ept1*Δ*/*Δ mutant do not differ significantly from those infected with wild type *C. albicans* (*n* = 10). The *n*-values for wild-type and *P_ENO1_-EPT1* were derived from three separate experiments, and those for *ept1*Δ*/*Δ were derived from two separate experiments. **(B)** Mice infected with *ept1*Δ*/*Δ have a decreased kidney fungal burden 5 days post infection compared to those infected with the wild type (^∗^*p* < 0.05; unpaired *t*-test, *n* = 15 mice per strain). Mice infected with the *ept1*Δ*/*Δ *P_ENO1_-EPT1* reintegrant were not significantly different than those infected with the wild type. Experiments were performed three times using five mice per strain and kidney homogenates were plated with two technical replicates for reproducibility. **(C)** Expression level of *C. albicans EPT1* affects cytotoxicity in co-culture with human epithelial cells *in vitro*. The *P_ENO1_-EPT1* overexpression strain has increased cytotoxicity (^∗^*p* < 0.05; unpaired *t*-test) compared to wild type, whereas *ept1*Δ*/*Δ has decreased cytotoxicity (^∗∗^*p* < 0.01). The *ept1*Δ*/*Δ *P_ENO1_-EPT1* reintegrant is not significantly different than wild type for cytotoxicity. Biological replicates were performed in triplicate and technical replicates were also performed in triplicate (*n* = 9 total replicates per strain).

Given that overexpression of *EPT1* increases PE/PC synthesis by the Kennedy pathway, we wanted to determine if this would also cause hypervirulence as observed for *pem1*Δ*/*Δ *pem2*Δ*/*Δ, which also accumulates PE (Figure 3). Therefore, wild-type and *P_ENO1_-EPT1* strains were tested in the mouse model of systemic candidiasis. Mice infected with *P_ENO1_-EPT1* *C. albicans* succumbed to infection more rapidly than those infected with wild type (^∗∗^*p* < 0.01) (Figure 7A). Thus, the *P_ENO1_-EPT1* and *pem1*Δ*/*Δ *pem2*Δ*/*Δ mutants both accumulate PE and exhibit hypervirulence.

We wanted to determine if this hypervirulence would correlate with increases in kidney fungal burden. Outbred ICR mice (Envigo) were infected with 5 × 10^5^ cells of the wild-type and *P_ENO1_-EPT1* strains. Five days post infection, the mice were necropsied and kidneys were harvested to determine fungal burden. Mice infected with the *P_ENO1_-EPT1* overexpression strain exhibited an average increase in fungal burden of 38.1% when compared to mice infected with wild type *C. albicans*, however, this difference was not statistically significant (Figure 7B, ^∗^*p* = 0.1885).

### Disruption of *EPT1* Results in a Decrease in Kidney Fungal Burden in Mice

As *EPT1* is necessary for PE and PC synthesis through the Kennedy pathway, and its overexpression increases virulence, we tested an *ept1*Δ*/*Δ mutant in the mouse model to determine if it exhibits decreased virulence. For survival curves in the systemic infection model there was not a significant decrease in virulence for the *ept1*Δ*/*Δ strain compared to wild type (Figure 7A). However, it should be noted that mice infected with the *ept1*Δ*/*Δ strain began to succumb to infection at 11 days post infection, which is slightly delayed compared to those infected with the wild-type (which begin to succumb to infection at 7 days post infection), which may indicate a modest, although not statistically significant, decrease in virulence. Although there was not a statistically significant difference in survival curves, mice infected with the *ept1*Δ*/*Δ mutant had a significant average decrease in kidney fungal burden of 44.97% as compared to the wild type (^∗^*p* < 0.05) (Figure 7B). This decrease in fungal burden may correlate with the slight delay seen in survival curves for mice infected with the *ept1*Δ*/*Δ strain. Conversely, mice infected with the reintegrant *ept1*Δ*/ept1*Δ*::P_ENO1_-EPT1* strain did not exhibit any significant differences in fungal burden compared to mice infected with wild type *C. albicans*.

### *EPT1* Expression Affects Cytotoxicity of *C. albicans* Against Epithelial Cells

The mechanism by which *EPT1* impacts virulence is unclear, as there are no clear impacts on growth rate in growth curves (data not shown). Thus, we examined another virulence factor, which is the ability of *C. albicans* to damage epithelial cells. This can be assessed by measuring the release of the cytoplasmic enzyme lactase dehydrogenase (LDH) from host cells. In order to measure cytotoxicity of each mutant compared to wild-type, 2.5 × 10^6^ *C. albicans* cells were co-cultured with human oral epithelial cells (FaDu cell line). A CytoTox 96^®^ cytotoxicity assay kit (Promega) was used to quantify the release of LDH after 4 h. There was a statistically significant decrease in cytotoxicity of the *ept1*Δ*/*Δ mutant as compared to the wild type (^∗∗^*p* < 0.01) (Figure 7C). Conversely, the *P_ENO1_-EPT1* overexpression mutant has increased cytotoxicity compared to the wild type (^∗^*p* < 0.05) (Figure 7C). The *ept1*Δ*/*Δ *P_ENO1_-EPT1* reintegrant does have a modest increase in cytotoxicity versus the wild type, but it is not statistically significant (Figure 7C).

## Discussion

In a previous communication we demonstrated that the synthesis of phospholipids via the CDP-DAG pathway is absolutely required for *Candida albicans* to be able to cause disease in the mouse model of systemic infection (Chen et al., 2010). The phospholipids produced in this pathway specifically are PS, PE, and PC (Figure 1). Blocking synthesis of PS or PE by disrupting the PS synthase (*cho1Δ/Δ* mutant) or both PS decarboxylases (*psd1Δ/Δ psd2Δ/Δ* mutant) results in avirulence (Chen et al., 2010). Although the full mechanism is not clear (Davis et al., 2018), PS and PE synthesis by the CDP-DAG pathway clearly play a crucial role in virulence. However, at the outset of this study the role of PE’s downstream product, PC, was less clear. In addition, no prior studies have directly evaluated the role of the Kennedy pathway with regard to virulence in *C. albicans*.

### Increased PE in Two Different Pathways Correlates With Hypervirulence

To determine if PC was important for the ability to cause disease, we blocked CDP-DAG mediated PC biosynthesis by the *pem1*Δ*/*Δ *pem2*Δ*/*Δ mutation. Systemic infections carried out in mice revealed that this mutation did not block virulence, but instead mice infected with the *pem1*Δ*/*Δ *pem2*Δ*/*Δ mutant succumb to infection more rapidly than those infected with wild-type (Figure 4).

The *pem1*Δ*/*Δ *pem2*Δ*/*Δ mutation causes a loss of PC synthesis by methylation of PE, but simultaneously leads to a build-up of PE (Figure 3). Synthesis of PE has been shown to be required for virulence (Chen et al., 2010; Davis et al., 2018), which suggests the possibility that increased PE might lead to greater virulence. Therefore, we increased PE synthesis through the Kennedy pathway by overexpressing *EPT1* (Figure 6), and this led to hypervirulence as well (Figure 7A). This suggests that increased PE synthesis causes hypervirulence, however, it should be noted that overexpression of *EPT1* also causes increased PC synthesis.

We favor increased synthesis of PE rather than PC as the explanation for hypervirulence since PE builds up, but PC goes down in the hypervirulent *pem1*Δ*/*Δ *pem2*Δ*/*Δ mutant (Figure 3). Thus, the common phospholipid to increase in both *pem1*Δ*/*Δ *pem2*Δ*/*Δ and *P_ENO1_-EPT1* (Figure 3, 6) is PE. Furthermore, PE synthesized from either the CDP-DAG or Kennedy pathways can support virulence (Chen et al., 2010; Davis et al., 2018). Thus, taken altogether, these data suggest PE is the primary phospholipid influencing virulence.

However, the mechanism by which elevated PE causes virulence is not clear at this point. We do note that increased *EPT1* correlates with increased damage of epithelial cells, and although modest, this assay is measuring only short term (4 h) damage, so increased damage over time (∼10 days) may accumulate leading to more rapid terminal infection. However, the full mechanism by which increased virulence is mediated is unknown.

### *EPT1* Is Required for Full Virulence During Systemic Infections

Overexpression of *EPT1* leads to hypervirulence, but we also wanted to determine whether the Kennedy pathway is required for full virulence in *C. albicans*, as this pathway is present in a variety of eukaryotic pathogens and is required for virulence in some (Gibellini et al., 2009). Furthermore, it has been demonstrated that blocking the ability of *C. albicans* to import glycerophosphocholine (GPC), an intermediate metabolite that may be shunted into the Kennedy pathway, attenuates virulence in the mouse model of systemic infection (Bishop et al., 2013). Until now, no virulence studies have been performed on the Kennedy pathway in *C. albicans*, and so it has also remained unknown whether this pathway is redundant in *C. albicans*, or if it is specifically required for growth in the host.

We found that disruption of *EPT1* caused a modest decrease in kidney fungal burden (Figure 7B), but not a significant increase in mouse survival (Figure 7A). This modest decrease in fungal burden might help explain the modest increase in the time in which mice begin to succumb to infection in the *ept1*Δ*/*Δ mutant (11 days) compared to wild-type (7 days) (Figure 7A), however, the overall difference in curves was not statistically significant, so the modest decrease in fungal burden does not appear to have a large impact on overall virulence. The decrease in kidney fungal burden also correlated with a modest decrease in damage to epithelial cells (Figure 7C). Thus, it is possible that the *ept1*Δ*/*Δ mutant is unable to damage host cells as well as wild-type *C. albicans*, and this could help explain the loss of fungal burden. Also, it is not entirely clear whether the effect is related to PE or PC, as Ept1 synthesizes both in *C. albicans*.

### *C. albicans* Ept1 Synthesizes Both PE and PC in the Kennedy Pathway

Prior to this study the model for the Kennedy pathway in *C. albicans* was based entirely on that elucidated for *S. cerevisiae*. However, we found that while all the enzymes involved in the first two steps of either PE or PC biosynthesis had homologs in *C. albicans*, only one enzyme for the last step of synthesis was found in *C. albicans*: Ept1. Given the results of the homology search and the fact that there is some overlap in substrate specificity between *EPT1* and *PCT1* in *S. cerevisiae* (Gibellini et al., 2009), we hypothesized that this enzyme was responsible for both PE and PC biosynthesis in *C. albicans.* This was tested using ^[14C]^ radiolabeling. Our findings are that the knockout cannot incorporate radiolabeled precursors into either PE or PC, and that when the gene is overexpressed an increased amount of the radiolabeled precursors are incorporated into PE and PC. This indicates that Ept1 is the only enzyme that catalyzes this reaction in *C. albicans*, and we can therefore block the Kennedy pathway by knocking out *EPT1* and increase phospholipid synthesis via this pathway by overexpressing *EPT1*.

We do not yet fully understand how the Kennedy pathway influences virulence, but the ability to modulate virulence in either direction using *EPT1* expression levels indicates that this is an important pathway for controlling the pathogenicity of this fungus.

## Author Contributions

RT, TR, and Y-LC participated in plasmid design and constructions, and *C. albicans* strain construction. RT performed mouse infection models with the assistance of WB or CC. RT and JD performed growth curves. SA and CC performed radiolabeling assays with direction from JP-V. RT performed all other work. TR directed the project and edited the manuscript. JP-V edited the manuscript as well.

## Conflict of Interest Statement

The authors declare that the research was conducted in the absence of any commercial or financial relationships that could be construed as a potential conflict of interest.
