# Supplementary material for: Overproduction of Phospholipids by the Kennedy Pathway Leads to Hypervirulence in Candida albicans
Source: Front Microbiol. 2019 Feb 7;10:86. doi: 10.3389/fmicb.2019.00086 (PMC6374345; doi:10.3389/fmicb.2019.00086)
Supplement: Supplementary file 2 [file Presentation_1.pdf]

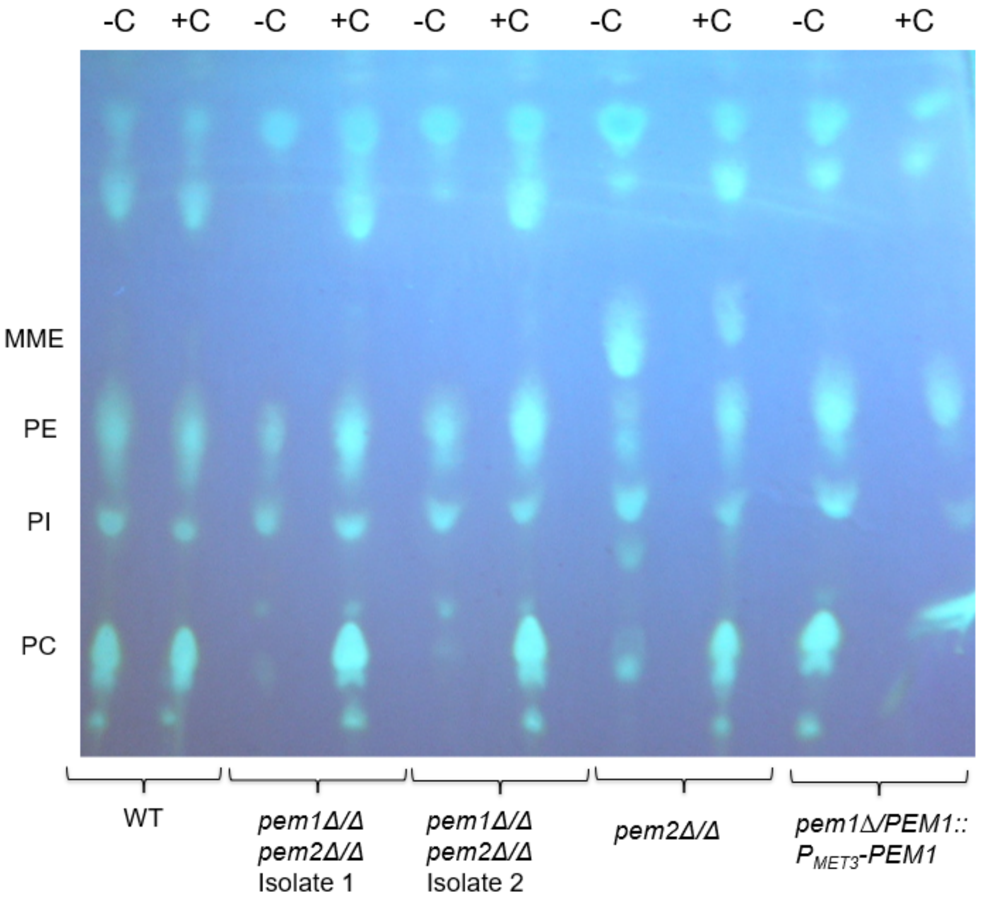

Figure S1. Choline auxotrophy correlates to a loss of PC. Wild-type, *pem1*Δ/Δ *pem2*Δ/Δ, *pem2*Δ/Δ, and *pem1*Δ/*pem1*::P_{MET3}-*PEM1* mutants were cultured in the presence of 0.25 mM L-methionine/cysteine in minimal media (YNB) with ("+C") or without ("-C") 1 mM choline and total lipids were extracted. Phospholipids were separated with thin layer chromatography (TLC) and visualized using primuline. The *pem1*Δ/Δ *pem2*Δ/Δ mutant only produces PC when supplemented with choline.
